# A p-Tyr42 RhoA Inhibitor Promotes the Regeneration of Human Corneal Endothelial Cells by Ameliorating Cellular Senescence

**DOI:** 10.3390/antiox12061186

**Published:** 2023-05-30

**Authors:** Hyeon Jung Kim, Jin Sun Hwang, Kyung Bo Noh, Sun-Hee Oh, Jae-Bong Park, Young Joo Shin

**Affiliations:** 1 Department of Ophthalmology, Hallym University Medical Center, Hallym University College of Medicine, Seoul 07442, Republic of Koreakylenoh@outlook.kr (K.B.N.); sunny5@hallym.ac.kr (S.-H.O.); 2 Hallym BioEyeTech Research Center, Hallym University College of Medicine, Seoul 07442, Republic of Korea; 3 Department of Biochemistry, Hallym University College of Medicine, Chuncheon 24252, Republic of Korea

**Keywords:** RhoA, human corneal endothelial cells, cell death, proliferation, TGF-β, oxidative stress

## Abstract

The development of treatment strategies for human corneal endothelial cells (hCECs) disease is necessary because hCECs do not regenerate in vivo due to the properties that are similar to senescence. This study is performed to investigate the role of a p-Tyr42 RhoA inhibitor (MH4, ELMED Inc., Chuncheon) in transforming growth factor-beta (TGF-β)- or H_2_O_2_-induced cellular senescence of hCECs. Cultured hCECs were treated with MH4. The cell shape, proliferation rate, and cell cycle phases were analyzed. Moreover, cell adhesion assays and immunofluorescence staining for F-actin, Ki-67, and E-cadherin were performed. Additionally, the cells were treated with TGF-β or H_2_O_2_ to induce senescence, and mitochondrial oxidative reactive oxygen species (ROS) levels, mitochondrial membrane potential, and NF-κB translocation were evaluated. LC3II/LC3I levels were determined using Western blotting to analyze autophagy. MH4 promotes hCEC proliferation, shifts the cell cycle, attenuates actin distribution, and increases E-cadherin expression. TGF-β and H_2_O_2_ induce senescence by increasing mitochondrial ROS levels and NF-κB translocation into the nucleus; however, this effect is attenuated by MH4. Moreover, TGF-β and H_2_O_2_ decrease the mitochondrial membrane potential and induce autophagy, while MH4 reverses these effects. In conclusion, MH4, a p-Tyr42 RhoA inhibitor, promotes the regeneration of hCECs and protects hCECs against TGF-β- and H_2_O_2_-induced senescence via the ROS/NF-κB/mitochondrial pathway.

## 1. Introduction

Human corneal endothelial cells (hCECs) are located on the innermost surface of the cornea and participate in the dehydration of the corneal stroma. They reportedly have limited proliferative capacity in vivo, although supplementation with various growth factors causes them to proliferate in vitro. Thus, efforts have been made to proliferate hCECs in vivo [1,2]. Rho-associated coiled-coil-containing protein kinase (ROCK) inhibitors promote the proliferation of corneal endothelial cells (CECs), although they inhibit proliferation of other cell types [3,4]. This is because ROCK inhibitors inhibit senescence [5,6]. Corneal endothelial cells (CECs) are different from vascular endothelial cells. CECs have a flattened hexagonal shape [7], while vascular endothelial cells have a spindle-like shape [8]. CECs are also found only in the cornea [7], while vascular endothelial cells are found throughout the body [9]. CECs do not express VE-cadherin, which has been known to be expressed in vascular endothelial cells [9]. CECs has been previously reported to express E-cadherin [10].

Transforming growth factor-beta (TGF-β) is a major cytokine that induces senescence [11], the process in which ROCK plays an important role [12]. Rho/ROCK signaling mediates TGF-β-induced actin organization and fibrosis via nuclear factor kappa-light-chain-enhancer of activated B cells (NF-κB) [13,14,15], where ROCK2 is particularly involved [15]. Moreover, TGF-β suppresses the proliferation of rabbit CECs in vitro [16], and inhibition of TGF-β signaling enables hCEC expansion in vitro for use in regenerative medicine [17], although it promotes cell barrier function upon maturation of CECs [18]. Furthermore, TGF-β receptor inhibitors suppress endothelial–mesenchymal transformation of hCECs [19]. TGF-β induces CEC senescence by increasing mitochondrial reactive oxygen species (ROS) levels [20]. Additionally, activation of TGF-β induces cell death via the unfolded protein response pathway in Fuchs’ endothelial corneal dystrophy [21].

The Rho/ROCK pathway is an important signaling pathway critically involved in cell proliferation, differentiation, migration, and apoptosis [22]. Rho is a member of the Rho subfamily of GTPases and includes RhoA, B, C, D, and E [23]. Rho proteins are regulated by various factors that control their activity and function within cells [23]. For example, guanine nucleotide exchange factor (GEF) leads to the release of GDP and binding of GTP to Rho proteins [24], causing their activation. GEFs are activated by several types of cell surface receptors including receptor tyrosine kinases, G-protein-coupled receptors, cytokines, and integrins [25]. ROCK is one of the most crucial downstream effectors of Rho [26] and belongs to the serine/threonine protein kinase family [26]. ROCK has two highly homologous isomers: ROCK1 and ROCK2 [27]. ROCK substrates vary depending on the tissue type [26]. ROCK1 is abundantly expressed in immunological cells, whereas ROCK2 is expressed in the brain and heart [28,29]. The function of ROCK1 and ROCK2 in modulating stress-induced actin dynamics, cell migration, and detachment is different [30], in that ROCK1 is involved in destabilizing actin cytoskeleton, whereas ROCK2 is involved in stabilizing actin cytoskeleton [30]. ROCK2 mediates cardiac hypertrophic responses [31,32], while ROCK1 participates in cardiac fibrosis [33]. ROCK1 is mainly activated by RhoA in its GTP-bound form, while ROCK2 is preferentially activated by p-Tyr42 RhoA in addition to the GTP-bound form [34]. In this study, we used a p-Tyr42 RhoA inhibitor as an inhibitor of the Rho/ROCK signaling pathway to induce the regeneration of hCECs. p-Tyr42 RhoA GTPase promotes reactive oxygen species (ROS) production via the phosphorylation of p47phox by ROCK and activates NF-κB [35,36].

ROCK2 is suspected to have a greater role in the proliferation of CECs and selective inhibition of ROCK2 via the inhibition of the p-Tyr42 RhoA pathway; the upstream signaling pathway of ROCK2 is expected to be the main mechanism of CEC proliferation and senescence. Thus, in this study, we investigated the role of a p-Tyr42 RhoA inhibitor in TGF-β-induced cellular senescence of hCECs.

## 2. Materials and Methods

### 2.1. Cell Culture

This study was performed in accordance with the tenets of the Declaration of Helsinki and reviewed and approved by the institutional review board/ethics committee of the Hallym University Medical Center. Human corneas were purchased from Eversight Eye Bank (Ann Arbor, MI, USA) and hCECs were cultured as previously described [37].

Cultured hCECs were treated with the p-Tyr42 RhoA inhibitor MH4 (10 µM; ELMED Inc., Chuncheon, Korea), TGF-β (10 ng/mL; ab50036, Abcam, Cambridge, MA, USA), or H_2_O_2_ (2 mM) in 5% CO_2_ incubator for 48 h. The hCECs were harvested for subsequent experiments. 

### 2.2. Cell Viability and Proliferation Assays

Cell counting kit-8 (CCK-8; Dojindo, Kumamoto, Japan) was used to assess cell viability. Briefly, cells (1 × 10^4^) were cultured in 96-well plates and treated with the CCK-8 reagents for 2 h. The cell viability was evaluated by measuring the optical density at 450 nm using a microplate reader (Synergy HTX, BioTek, Winooski, VT, USA) [38].

Bromodeoxyuridine (BrdU) proliferation assay kit (Roche Diagnostics, GmbH, Mannheim, Germany) was used to evaluate the cell proliferation rate according to the manufacturer’s protocol. Cells (5 × 10^3^ cells/well) were placed in 96-well plates and incorporated with BrdU at 37 °C and 5% CO_2_. After incubating the cells with FixDenat solution for 30 min at 25 °C, the cells were treated with anti-BrdU-POD reagents for 90 min at 25 °C. The substrate reagents were put into each well, and incubated for 15 min at 25 °C. Then, 1 M H_2_SO_4_ solution was put into each well. Absorbance was evaluated at 450 nm using a microplate reader (Synergy HTX, BioTek) [39]. Proliferation rates are determined as the fold of controls.

Cytotoxicity was assessed using a lactate dehydrogenase (LDH) cytotoxicity detection assay (MK401, Takara Bio Inc., Shiga, Japan). Briefly, cell culture supernatants containing LDH released from damaged cells were obtained, added to 96-well plates, and incubated for 10–30 min with the reaction mix. Absorbance was assessed at 490 nm using a multi-mode microplate reader (Synergy HTX, BioTek) [40].

To construct a growth curve, cells were incubated under controlled conditions. At regular intervals, the number of cells is determined by counting using a microscope at three low-power fields (×5).

### 2.3. Cell Cycle Analysis

Cell cycle was analyzed using flow cytometry (CytoFLEX, Beckman Coulter Life Sciences, Miami, FL, USA) and propidium iodide (PI) reagent. The number of cells in each phase was compared.

### 2.4. Cell Adhesion Assay

Cell adhesion was evaluated for 2 or 6 h. Confluent cultured cells were pretreated with 10 μM MH4 for 30 min. The cells were trypsinized, resuspended in the corresponding culture medium, and standardizing to an equal cell number of 2 × 10^5^/mL. The equal number of cells (50 µL) was put into each well of 96-well plates. Cells were allowed to adhere to the bottom of plates for 2 or 6 h, and non-adherent cells were rinsed off with PBS. Then, 4% paraformaldehyde was used for the fixation of cells for 30 min at 25 °C and stained with 0.1% crystal violet solution for 1 h. The culture dishes were extensively rinsed with distilled water to draw off excess dye, and the stain was dissolved with 10% acetic acid. Optical density at 570 nm was determined using a multi-mode spectrophotometer.

### 2.5. Immunofluorescence Staining

hCECs were cultured on cell culture slides (SPL Life Sciences, Seoul, Korea), washed with PBS, and fixed using 4% paraformaldehyde for 30 min. Permeabilization was performed for 10 min using 0.5% Triton X-100 and blocking was performed for 60 min using 1% skim milk at 25 °C. Then, the cells were treated with mouse anti-human Ki-67 antibody (sc-23900; Santa Cruz Biotechnology, Santa Cruz, CA, USA), mouse anti-human E-cadherin antibody (sc-8426; Santa Cruz Biotechnology), or rabbit anti-human NF-κB antibody (sc-372; Santa Cruz Biotechnology) at 4 °C, followed by rinsed with PBS. The cells were treated with either fluorescein isothiocyanate-conjugated goat anti-rabbit IgG antibody (1:100) for 2 h at 25 °C and the nucleus were stained with Hoechst 33342 reagent (1:2000; Molecular Probes, Eugene, OR, USA). The cells were evaluated under a fluorescence microscope (DMi8, Leica Microsystems, Hesse, Germany) and photographs were captured.

hCEC adhesion was assessed using the actin staining marker phalloidin. F-actin was stained using Alexa Fluor 488H phalloidin (Molecular Probes) and Hoechst 33342 nuclear stain, and cells were evaluated under a fluorescence microscope (DMi8; Leica Microsystems).

### 2.6. Western Blotting

Radioimmunoprecipitation assay buffer (Biosesang, Seoul, Korea) including phosphatase (PhosSTOP; Roche, Basel, Switzerland) inhibitor cocktails and protease (Sigma-Aldrich, St. Louis, MO, USA) was employed to obtain total proteins. Western blot was conducted by standard methods. Briefly, 5% skim milk was added for 1 h to block nonspecific binding. The primary antibodies used were: mouse anti-ROCK1 antibody (sc-17794, Santa Cruz Biotechnology, 1:500 dilution), mouse anti-ROCK2 antibody (sc-398519, Santa Cruz Biotechnology, 1:500 dilution), mouse anti-LC3 antibody (M186-3, MBL, Fujioka-Shi, 1:1000 dilution), and rabbit anti-GAPDH antibody (LF-PA0212, Abfrontier,Seoul, 1∶5000 dilution). A horseradish peroxidase-conjugated secondary antibody and WEST-Queen™ Western Blot Detection Kit (iNtRON Biotechnology, Seongnam, Korea) were employed. Video image analysis was used to quantify the immunoreactive band.

To evaluate NF-kB levels in nucleus and cytoplasm, NE-PER nuclear and cytoplasmic extraction reagents (Thermo Fisher Scientific Inc., Waltham, MA, USA) was used. Cells were harvested, rinsed with PBS, and centrifuged at 200× *g* for 5 min. After nuclear proteins were extracted, cytoplasmic extraction reagent I including phosphatase and protease inhibitor cocktails (1:100) was put into the samples for 10 min on ice. Cytoplasmic extraction reagent II was put into the samples for 1 min. Samples were centrifuged at 16,000× *g* for 5 min at 4  °C. Then, Western blotting was performed. Primary antibodies were as follows: rabbit anti-human NF-κB antibody (sc-372; Santa Cruz Biotechnology), β-actin (LF-PA0207, Abfrontier, Seoul, 1:5000 dilution) and rabbit anti-GAPDH antibody (LF-PA0212, Abfrontier, Seoul, 1:5000 dilution).

### 2.7. Senescence-β-Galactosidase Assay and Cell Size Measurements

Senescence-β-galactosidase staining kit (BioVision) was used for senescence-β-galactosidase (SA-β-gal) staining. Briefly, after eliminating the growth medium, cells were washed with PBS. Each well was incubated with a fixative solution for 10–15 min at RT to fix the cells. After rinsing the cells with PBS, they were treated with β-galactosidase staining reagent at 37 °C overnight in a dry incubator.

To quantify the changes of cell shape, AxioVision Rel. 4.7 software (Carl Zeiss Meditec, Oberkochen, Germany) was used to outline the boundaries of cells and to measure the area of each cell in pixels. The measured areas were then represented as fold change, which is a measure of the relative difference between two values.

### 2.8. Mitochondrial Oxidative Stress Evaluation

MitoSOX^TM^ Red (Invitrogen) was used according to the manufacturer’s protocol to measure mitochondrial superoxide production. Cells were treated with 5 μM MitoSOX^TM^ solution for 20 min at 37 °C in the dark. Cytoflex analyzer (Beckman Coulter Life Sciences) was used to measure the fluorescence intensity at an excitation wavelength of 510 nm and emission wavelength of 590 nm.

### 2.9. Mitochondrial Membrane Potential Assay

Mitochondrial membrane potential was assessed using Muse™ MitoPotential assay kit (Merck Millipore, Guyancourt, France). The MitoPotential dye was employed to indicate the mitochondrial membrane potential, and 7-AAD was used to indicate cell death. Cytoflex flow cytometer (Beckman-Coulter, Brea, CA, USA) was used to analyze the data. JC-1 at a final concentration of 2.5 μM was used for fluorescence microscopy to determine the mitochondrial membrane potential. 

### 2.10. Real-Time Reverse Transcription-Polymerase Chain Reaction (qRT-PCR)

RNA was extracted from the cultured hCEnCs separately using the ReliaPrep™ RNA Miniprep Systems (Promega, Madison, WI, USA) [41]. First-strand cDNA was synthesized from 200 ng of oligonucleotide primers using a commercially available kit (GoScript Reverse Transcription System; Promega). Real-time quantification of transcripts was performed on a LightCycler^®^ 96 (Roche Life Science, Mannheim, Germany) using the AccuPower 2X GreenStar qPCR Master Mix (Bioneer). The data were expressed as fold-changes relative to the control. The comparative CT or ΔΔCT methods was used to conduct relative quantification. After normalization of the target gene to the reference gene, β-actin, the expression level of the target gene was presented as the relative change from the control treatment. The primers are described in Supplementary Table S1.

### 2.11. Transcriptome Analysis

Transcriptome was analyzed in order to investigate the effect of MH4 on gene expression patterns, regulatory mechanisms, and functional pathways within a biological system. RNA was extracted from cells treated with or without MH4. RNA sequencing was conducted by MacroGen Inc. (Seoul, Republic of Korea, www.macrogen.com, accessed on 18 December 2022). Quant-IT RiboGreen (Invitrogen, #R11490) was used to measure total RNA amount. The samples are run on the TapeStation RNA screentape (Agilent, #5067-5576) for evaluation of the integrity of the total RNA. Only high-quality RNA preparations, with RIN greater than 7.0, were employed for RNA library construction.

Illumina TruSeq Stranded mRNA Sample Prep Kit (Illumina, Inc., San Diego, CA, USA, #RS-122-2101) was used to prepare a library with 1 ug of total RNA independently. Purification of the poly-A-containing mRNA molecules was performed using poly-T-attached magnetic beads. Then, the mRNA was fragmented into small pieces using divalent cations under elevated temperature. SuperScript II reverse transcriptase (Invitrogen, #18064014) and random primers was used to copy the cleaved RNA fragments into first-strand cDNA. Then, second-strand cDNA synthesis using DNA polymerase I, RNase H, and dUTP was conducted. The cDNA fragments went through an end repair process, the addition of a single ‘A’ base, and ligation of the adapters. The products were purged and enriched with PCR to create the final cDNA library.

KAPA library quantification kits for Illumina Sequencing platforms according to the qPCR quantification protocol guide (KAPA BIOSYSTEMS, #KK4854) was used to quantify the libraries. TapeStation D1000 ScreenTape (Agilent Technologies, # 5067-5582) was used to qualify the libraries. The paired-end (2 × 100 bp) sequencing was performed by an Illumina NovaSeq (Illumina, Inc., San Diego, CA, USA).

FastQC v 0.11.7 (http://www.bioinformatics.babraham.ac.uk/projects/fastqc/, accessed on 11 September 2022) was used to evaluate quality control test of RNA-seq [42]. To eliminate 3′ end adaptors and low-quality bases, Trimmomatic 0.38 (http://www.usadellab.org/cms/?page=trimmomatic, accessed on 11 September 2022) was used [43]. They were trimmed and then cleaned reads were aligned to the human reference genome (hg19) acquired from the University of California Santa Cruz genome browser by HISAT2 version 2.1.0 software (https://ccb.jhu.edu/software/hisat2/index.shtml, accessed on 11 September 2022) [44].

### 2.12. Analysis of Differentially Expressed Genes (DEGs) and Functional Analyses of DEGs

To calculate transcript abundances and affirm differentially expressed genes (DEGs) between treated and control groups, StringTie version 1.3.4d (https://ccb.jhu.edu/software/stringtie/, accessed on 14 September 2022) and DESeq2 software were employed [45,46]. Transcript abundance was computed by fragments per kilobase of transcripts per million mapped reads (FPKM). DEGs were determined as genes satisfying the fold change (FC) ≥ 2 and a raw *p*-value < 0.05 between groups. False discovery rate (FDR) control was performed by adjusting *p*-value calculated from Benjamini–Hochberg algorithm.

DEGs were aligned into the Gene Ontology (GO) databases to investigate the biological functions of DEGs. The q-value ≤ 0.05 calculated after the *p*-value was adjusted by multiple *t*-tests was employed as the threshold, and the GO term and path that were significantly enriched in the DEGs were investigated. GO enrichment analysis was conducted to classify DEGs based on specific biological functions using a ToppGene (https://toppgene.cchmc.org/enrichment.jsp, accessed on 18 May 2023) [47]. Molecular function of GO categories from ToppGene was presented. GO terms and pathways satisfying adjusted *p*-value < 0.05 were considered significantly enriched.

### 2.13. Autophagy Detection Imaging

CYTO-ID^®^ Autophagy detection kit (ENZO) was used to monitor autophagy in live cells by fluorescent microscopy [48]. Briefly, cells were rinsed with PBS and incubated in Cyto-ID staining solution at 37 °C for 30 min in the dark. Cells were rinsed twice with PBS to eliminate the free dyes. Hoechst 33342 nuclear staining was performed, and cells were observed under a fluorescence microscope (DMi8; Leica Microsystems).

### 2.14. Statistical Analysis

Data were presented as mean ± standard deviation. GraphPad Prism v.9 (GraphPad Software, San Diego, CA, USA) was used for statistical analysis. An independent *t*-test was used to compare two groups. One-way analysis of variance (ANOVA) followed by Tukey’s multiple comparison test was used for more than two groups. All experiments were repeated more than three times. The individual values are presented in Figures, which show the number of repeats. The number of samples was decided using a two-sided test with α = 0.05 and β = 0.2 (80% power).

## 3. Results

### 3.1. MH4 Increases the Viability and Proliferation of Cultured hCECs

**Figure 1:**
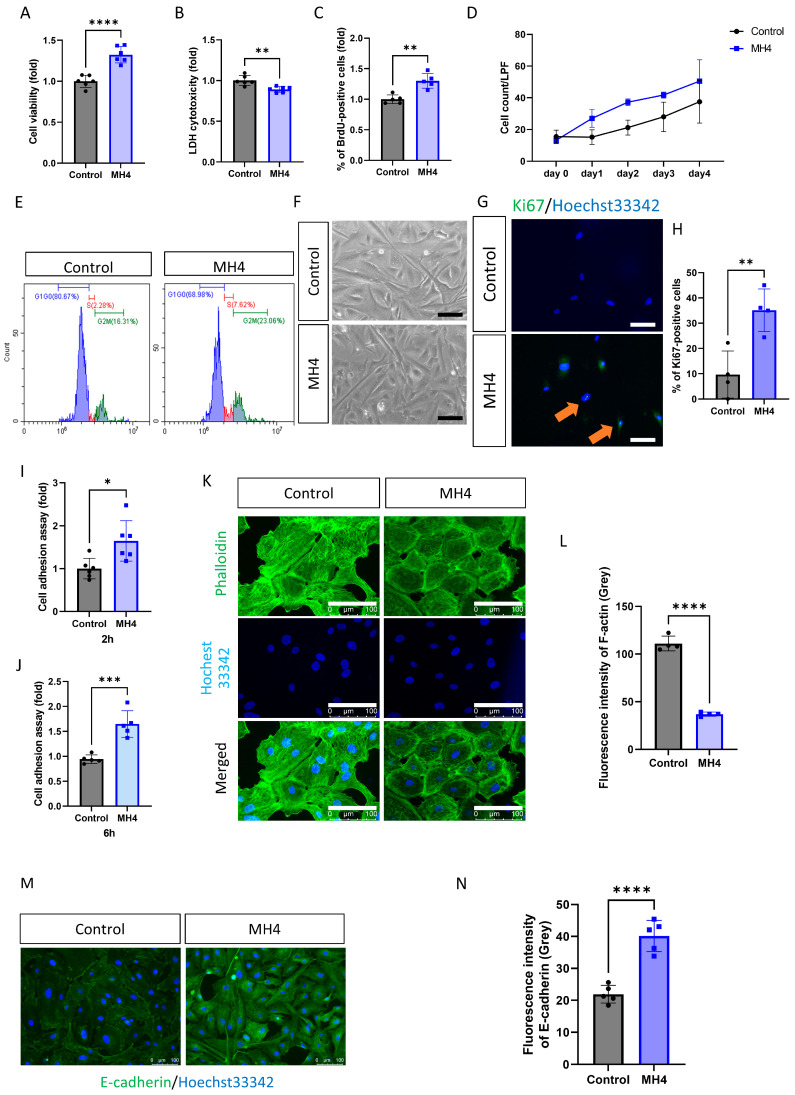
MH4 on proliferation of hCECs. (**A**) CCK-8 was employed to measure cell viability and (**B**) LDH assay was used to evaluate cytotoxicity. Proliferation rate was evaluated using BrdU proliferation assay (**C**), cell growth curve (**D**), cell cycle analysis (**E**), cell shape (**F**), and immunofluorescence staining of Ki67 (**G**,**H**). scale bar = 100 μm. (**I**,**J**) Cell adhesion assay was performed at 2 h and 6 h using crystal violet assay. (**K**,**L**) Actin filaments arrangement was assessed by phalloidin. (**M**,**N**) Immunofluorescence staining of E-cadherin was performed. E-cadherin (green) and nucleus (blue) was observed. Scale bar = 100 μm. * *p* < 0.05, ** *p* < 0.01, *** *p* < 0.001 and **** *p* < 0.0001 by Student’s *t*-test.

To evaluate the effect of MH4 on the health and function of cells, cell viability and proliferation rate were assessed. Cell viability is elevated and cytotoxicity is reduced by MH4 (Figure 1A,B). Moreover, MH4 elevates the cell proliferation rate (Figure 1C,D) and shifts the cells to the S phase (Figure 1E). The cells are small and less slender (Figure 1F). Immunofluorescence staining for Ki-67, a proliferation marker, shows that MH4 increases the number of Ki-67-positive cells (Figure 1G,H). Similarly, cell adhesion is increased by MH4 (Figure 1I,J). F-actin expression is decreased and E-cadherin expression is increased by MH4 (Figure 1K–N).

### 3.2. MH4 Affects Transcriptome

**Figure 2:**
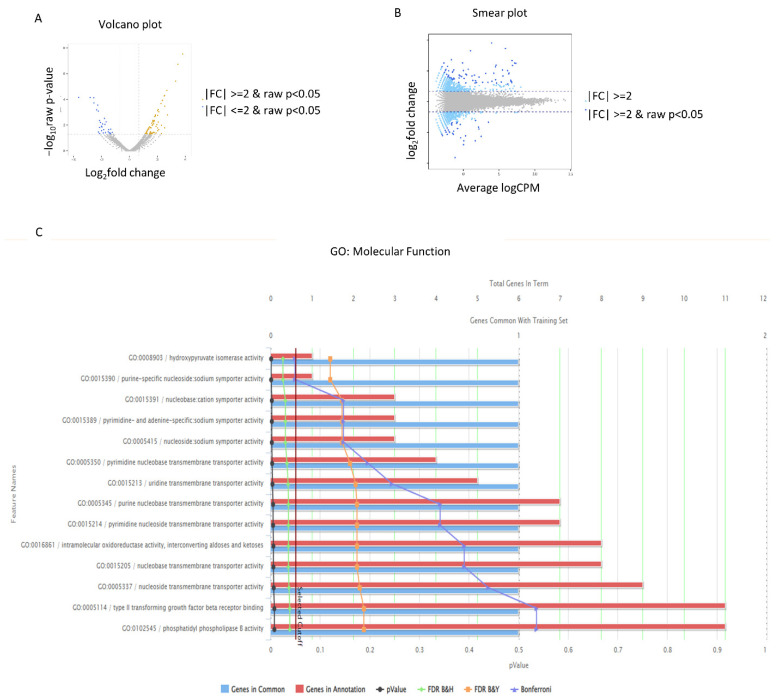
MH4 on proliferation of hCECs. (**A**,**B**) Volcano plot and smear plot are shown. (**C**) Molecular function of Gene Ontology is shown.

Transcriptome analysis was performed using NGS sequencing in order to identify differentially expressed genes (DEGs) and explore the gene expression patterns in a specific biological sample or condition [49]. Transcriptome analysis provides valuable insights into the overall gene expression profile and allows for the identification of genes that are upregulated or downregulated in response to MH4 [49]. The DEGs of MH4-treated cells are described in Table 1. A volcano plot and smear diagram of DEG levels are shown in Figure 2A,B. Molecular function of Gene Ontology is shown in Figure 2C and Table 2. TGFBRII signaling, phosphatidyl phospholipase B activity, and nucleobase transmembrane transporter activity are involved. 

### 3.3. MH4 Protects the Cultured hCECs against TGF-β-Induced Senescence

**Figure 3:**
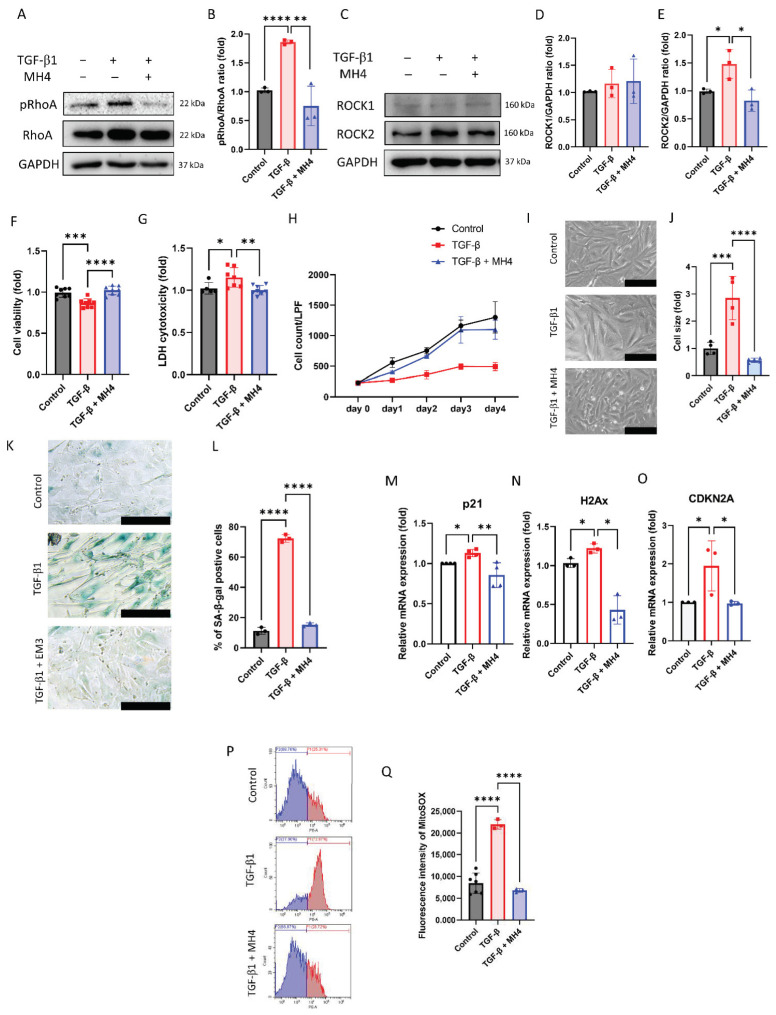
Effect of MH4 on TGF-β-induced senescence. (**A**,**B**) pRhoA and RhoA levels are evaluated using Western blot. (**C**,**D**,**E** ROCK1 and ROCK2 levels are evaluated using Western blot. (**F**) Cell viability is conducted by CCK-8. (**G**) LDH cytotoxicity. (**H**) Comparison of cell growth curve in cells with or without TGF-β or MH4. (**I**,**J**) Cell shape and cell size are evaluated by inverted microscopy. Scale bar = 250 μm. (**K**,**L**) Senescence-β-galactosidase staining is conducted and blue indicates the positivity of senescence-β-galactosidase. Scale bar = 100 μm. (**M**–**O**) mRNA expressions of *p21, H2Ax,* and *CDKN2A* are measured using RT-qPCR. (**P**,**Q**) Mitochondrial oxidative stress levels are measured using MitoSOX probe. Data are expressed as mean ± S.D. * *p* < 0.05, ** *p* < 0.01, *** *p* < 0.001, and **** *p* < 0.0001 by ANOVA, followed by Tukey’s test.

To evaluate the effect of MH4 on TGF-β-induced senescence, RhoA, ROCK1, and ROCK2 levels were evaluated as the non-canonical pathway of the TGF-β signaling pathway. TGF-β elevates pRhoA and ROCK2 levels (Figure 3A,B), which are attenuated by MH4. TGF-β decreases cell viability and increases LDH cytotoxicity; however, these effects are reversed by MH4 (Figure 3C,D). To evaluate the effect of MH4 on TGF-β-induced senescence, cell growth curve analysis was conducted. Cell growth curve reveals the growth arrest in TGF-β-treated cells, which is ameliorated by MH4 (Figure 3E). The cell size increases after TGF-β treatment, which is attenuated by MH4 (Figure 3F,G). Furthermore, TGF-β increases the percentage of SA-β-gal-positive cells (Figure 3H,I), and mRNA expressions of *p21, H2Ax,* and *CDKN2A*, which is reversed by MH4 (Figure 3J–L). Intracellular oxidative stress levels are elevated by TGF-β, which is attenuated by MH4 (Figure 3M,N).

**Figure 4:**
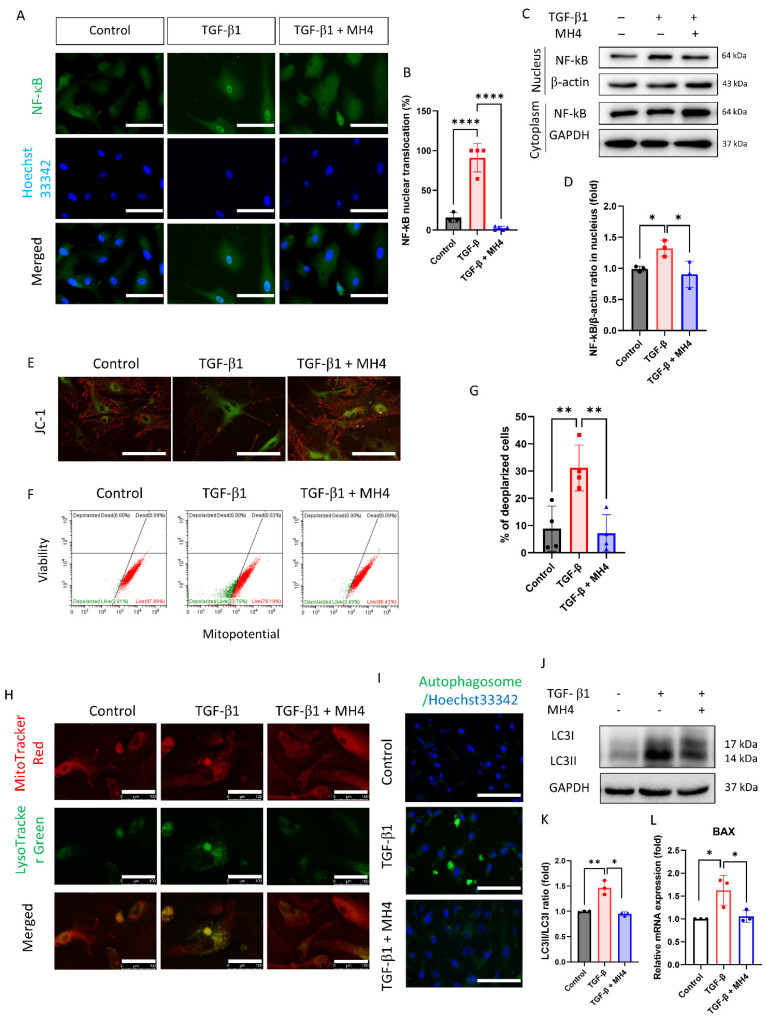
Effect of MH4 on TGF-β-induced mitochondrial membrane potential depolarization and autophagy. (**A**–**D**) Nuclear factor-κB (NF-κB) nuclear translocation. Scale bar = 100 μm. (**E**) JC-1 probe is employed to obtain the images of mitochondrial membrane potential. Scale bar = 100 μm. (**F**,**G**) MitoPotential kit is used to evaluate mitochondrial membrane potential depolarization. (**H**) Lysosomes are evaluated using LysoTracker green. Scale bar = 100 μm. (**I**) Autophagosome is demonstrated using autophagy detection kit. Scale bar = 100 μm. (**J**,**K**) LC3II is evaluated by Western blotting. (**L**) mRNA expressions of *BAX* are evaluated by RT-qPCR. Data are expressed as mean ± S.D. * *p* < 0.05, ** *p* < 0.01, and **** *p* < 0.0001 by ANOVA, followed by Tukey’s test.

To evaluate the effect of MH4 on the senescence-associated pathway, NF-kB signaling was assessed. TGF-β promotes the nuclear translocation of NF-kB (Figure 4A–C). NF-kB activation is linked to mitochondrial dysfunction [50]. Thus, mitochondrial membrane potential was assessed. TGF-β decreases the mitochondrial membrane potential, while MH4 ameliorates the mitochondrial membrane potential (Figure 4D–F). Lysosomes are more prominent and autophagy increases after TGF-β treatment, which is one of the feature of senescent cells [51]; however, these effects are ameliorated by MH4 (Figure 4G,H). LC3II levels, a marker of autophagy [52], are elevated after TGF-β treatment, but suppressed by MH4 (Figure 4I). Furthermore, *BAX* mRNA expressions are elevated after TGF-β treatment, but suppressed by MH4 (Figure 4J). BAX is required for autophagy [53].

### 3.4. MH4 Protects the Cultured hCECs against Oxidative-Stress-Induced Senescence

**Figure 5:**
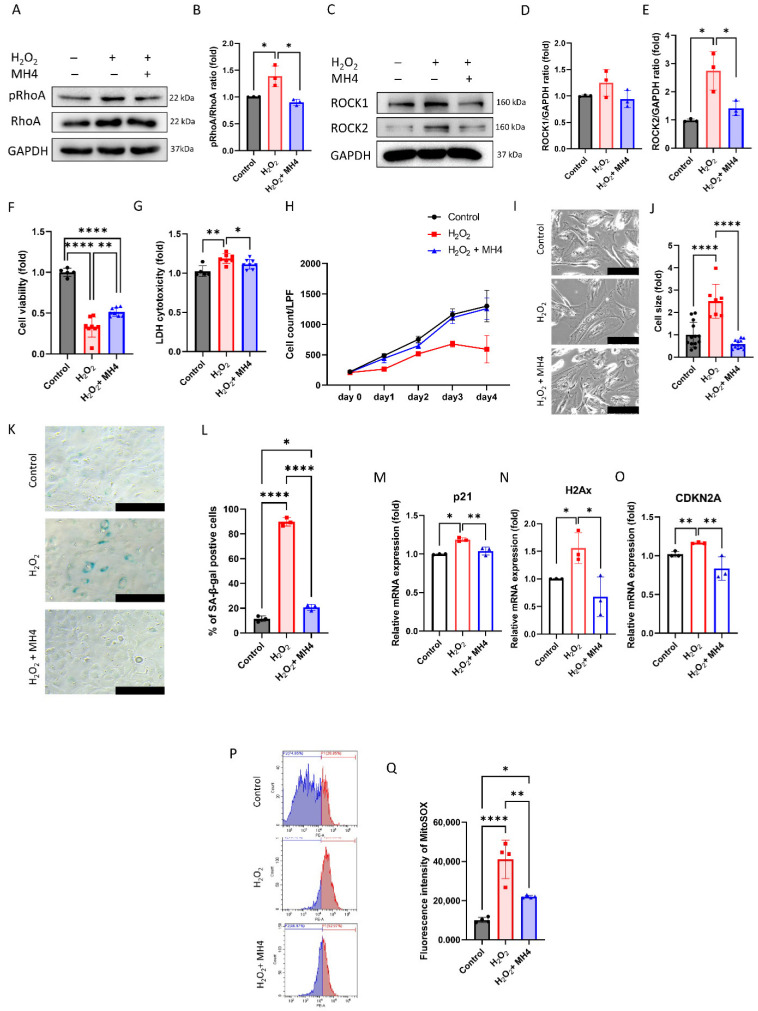
Effect of MH4 on H_2_O_2_-induced senescence. (**A**,**B**) pRhoA and RhoA levels evaluated using Western blot. (**C**–**E**) ROCK1 and ROCK2 levels evaluated using Western blot. (**F**) Cell viability by CCK-8 assay. (**G**) LDH cytotoxicity. (**H**) Comparison of cell growth curve in cells with or without H_2_O_2_ or MH4. (**I**,**J**) Cell shape and cell size evaluated by inverted microscopy. Scale bar = 100 μm (**K**,**L**) Senescence-β-galactosidase staining is conducted and blue indicates the positivity of senescence-β-galactosidase. Scale bar = 100 μm. (**M**–**O**) mRNA expressions of *p21, H2Ax,* and *CDKN2A* measured using RT-qPCR. (**P**,**Q**) Mitochondrial oxidative stress levels measured using MitoSOX probe. Data are expressed as mean ± S.D. * *p* < 0.05, ** *p* < 0.01, and **** *p* < 0.0001 by ANOVA, followed by Tukey’s test.

H_2_O_2_ has been reported to modulate TGF-β signaling transduction and H_2_O_2_ is generated in TGF-β signaling transduction [54]. Thus, we evaluated whether MH4 could protect the HCECs against H_2_O_2_-induced senescence. H_2_O_2_ treatment elevates pRhoA and ROCK2 levels, which are members of the non-canonical pathway of TGF-β and attenuated by MH4 (Figure 5A,B). H_2_O_2_ treatment decreases the cell viability and increases LDH cytotoxicity, both of which are ameliorated by MH4 (Figure 5C,D). The cell growth curve reveals the growth arrest, a hallmark of senescence, in H_2_O_2_-treated cells, which is ameliorated by MH4 (Figure 5E). Moreover, the cell size is increased by H_2_O_2_, but decreased by MH4 (Figure 5F,G). Similarly, the percentage of SA-β-gal-positive cells is elevated by H_2_O_2_ treatment, but reduced by MH4 (Figure 5H,I). mRNA expressions of *p21, H2Ax*, and *CDKN2A* are increased by H_2_O_2_ treatment, which is ameliorated by MH4 (Figure 5J–L). Intracellular oxidative stress levels are increased by H_2_O_2_ treatment, but reduced by MH4 (Figure 5H,I).

**Figure 6:**
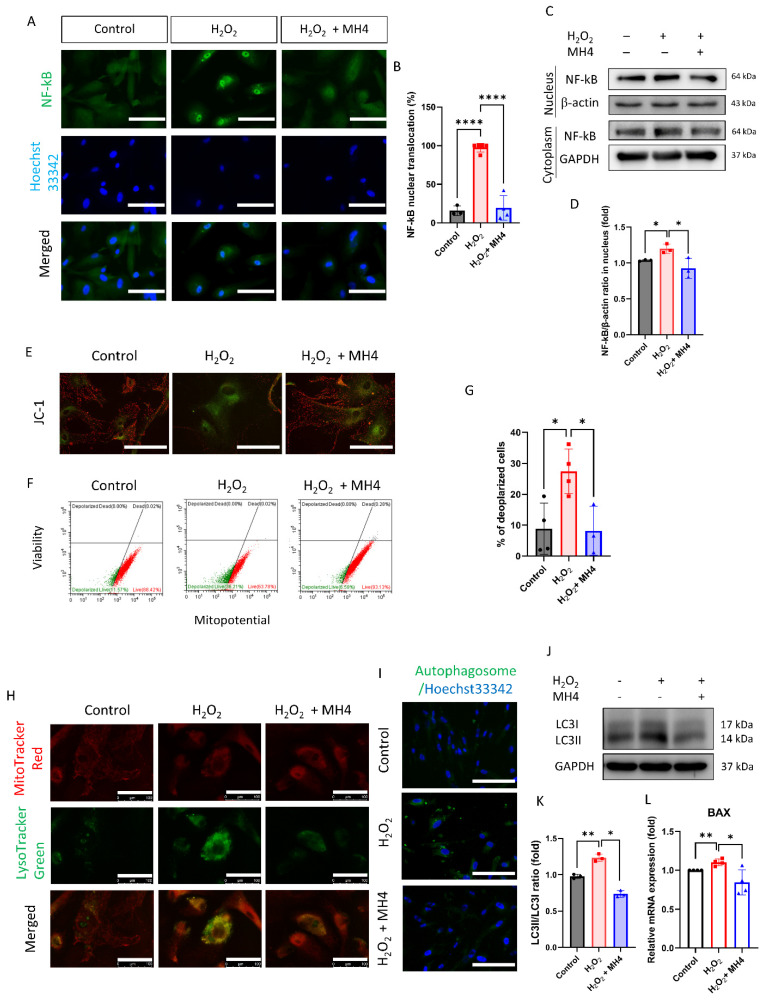
Effect of MH4 on H_2_O_2_-induced mitochondrial membrane potential depolarization and autophagy. (**A**–**D**) Nuclear factor-κB (NF-κB) nuclear translocation. Scale bar = 100 μm. (**E**) JC-1 probe used to evaluate the mitochondrial membrane potential. Scale bar = 100 μm. (**F**,**G**) MitoPotential kit used to evaluate mitochondrial membrane potential depolarization. (**H**) Lysosome evaluated using LysoTracker green. Scale bar = 100 μm. (**I**) Autophagosome is demonstrated using autophagy detection kit. Scale bar = 100 μm. (**J**,**K**) LC3II evaluated by Western blotting. (**L**) mRNA expressions of *BAX* evaluated by RT-qPCR. Data are expressed as mean ± S.D. * *p* < 0.05, ** *p* < 0.01, and **** *p* < 0.0001 by ANOVA, followed by Tukey’s test.

Furthermore, we evaluated the effect of MH4 on H_2_O_2_-induced cellular pathologic responses. H_2_O_2_ treatment promotes the nuclear translocation of NF-κB, which is attenuated by MH4 (Figure 6A–C). H_2_O_2_ decreases the mitochondrial membrane potential, while MH4 reverses this effect (Figure 6D–F). H_2_O_2_ increases lysosomes and autophagy, but is ameliorated by MH4 (Figure 6G,H). LC3II levels, a marker of autophagy, are elevated by H_2_O_2_, but suppressed by MH4 (Figure 6I). Additionally, *BAX* mRNA expressions are elevated by H_2_O_2_, but suppressed by MH4 (Figure 6J).

## 4. Discussion

Corneal endothelial cells (CECs), which are located at the innermost layer of the cornea and dehydrate the corneal stroma, are different from vascular endothelial cells [55]. CECs originate from neural crest cells [55]. TGF-β is a multifunctional cytokine that plays a crucial role in regulating cell growth, differentiation, migration, and survival [20,56]. In many cell types, TGF-β signaling can inhibit cell proliferation including in hCECs through Rho/ROCK signaling [3,20]. Rho/ROCK signaling is a pathway that regulates actin cytoskeleton dynamics, cell proliferation, migration, and adhesion [57]. Inhibition of Rho/ROCK signaling has been shown to promote hCEC proliferation [58]. TGF-β binds to the TGF-β receptor, and then TGFBRII activates RhoA, which subsequently activates ROCK1 and ROCK2 [59]. It has been suggested that p-Tyr42 on RhoA may play a role in regulating TGF-β signaling and its downstream effects on transcription regulation [59]. In this study, we reveal that MH4, a p-Tyr42 RhoA inhibitor, promotes the regeneration of hCECs. ROCK inhibitors, such as Y27632 and fasudil, promote the proliferation of hCECs [58,60,61] by facilitating the degradation of p27Kip1 (p27), promoting the cyclin D levels [61], and inducing the loss of actin stress fibers and cell–cell tight junctions [58]. The shape of CECs is restored after treating with ROCK inhibitors [58]. p-Tyr42 RhoA is a protein upstream of ROCK [35]. p-Tyr42 RhoA inhibition selectively suppresses the activated form of RhoA and, subsequently, inhibits ROCK2 [35], which is necessary for the stabilization of the actin cytoskeleton by modulating cofilin phosphorylation [30]. ROCK2 is a preferential activated by p-Tyr42 RhoA [34]. F-actin attenuation is associated with proliferation in CECs different from vascular endothelial cells [58]. F-actin filaments are involved in the formation of cellular protrusions called filopodia, which are important for cell migration and the formation of new blood vessels [62].

Transcriptome analysis shows that MH4 have an effect on TGF-β signaling, hydroxypyruvate isomerase, and nuclear transmembrane transporters. Hydroxypyruvate isomerase is an enzyme involved in the conversion of hydroxypyruvate to pyruvate, a key step in energy metabolism [63]. Changes in hydroxypyruvate isomerase activity may impact cellular energy production and metabolic pathways. Nuclear transmembrane transporters are responsible for the movement of molecules across the nuclear envelope, regulating the exchange of proteins, nucleic acids, and other molecules between the cytoplasm and the nucleus [64]. Changes in the activity of nuclear transmembrane transporters can have broad implications for cellular processes such as gene expression, DNA replication, and cellular signaling [65,66]. MH4 may translocate the signaling molecules across the nuclear transmembrane into the nucleus via these nuclear transmembrane transporters.

To investigate whether the failure of cells to regenerate is due to senescence or failure to proliferation, the number of cultured cells overtime was assessed and cell growth curve was added. It is confirmed that cell growth stops when treated with TGF-β and H_2_O_2_, which is regarded as senescence. CECs undergo senescence with age, which is involved in CDKN2A and p53 signaling pathways [20,67,68]. TGF-β has been reported to induce senescence in CECs [20,56]. In this study, MH4 suppresses TGF-β-induced senescence by regulating the ROS/NF-κB signaling pathway. TGF-β accelerates or causes senescence and senescence-related phenotypes in a variety of cells [11,20]. A variety of signaling pathways, including ROS, p53, and NF-κB, are involved in TGF-β-induced senescence [69,70,71]. TGF-β promotes ROS production in the mitochondria in several cell types [72,73]. ROS modify histones and DNA by participating in interconnected epigenetic phases and contribute to the onset and progression of cellular senescence by directly damaging mtDNA [74]. TGF-β signaling can activate the Rho/ROCK pathway linking NF-κB translocation into the nucleus [75,76]. NF-κB is activated during senescence and induces the secretion of senescence-associated secretory phenotype (SASP) factors, which are mainly inflammatory cytokines [71].

This study reveals that MH4 ameliorates TGF-β-induced mitochondrial depolarization and autophagy. The depolarization of mitochondrial membrane potential is linked to mitochondrial dysfunction because the maintenance of the membrane potential is required to generate energy in the mitochondria [77]. Loss of mitochondrial membrane potential results in a deprivation of cellular energy, reducing the cell viability, and causing cell death [78]. Autophagy is considered a cell survival and death mechanism and regulates the number and health of mitochondria [79]. Mitochondrial proteins are degraded by autophagy in aged cells [80]. Although autophagy redeems defects in mitochondrial dynamics by blocking mitochondrial fission, mitochondrial autophagy is induced for selective clearance of damaged mitochondria in cells [81,82]. TGF-β induces depolarization of mitochondrial membrane potential and autophagy by elevating mitochondrial ROS levels [83,84].

As TGF-β increases ROS in hCECs, we investigate whether MH4 attenuates the effect of oxidative stress. Oxidative stress has been reported as a major cause of senescence [85,86]. In this study, MH4 inhibits H_2_O_2_-induced senescence by regulating the ROS/NF-κB signaling pathway. Exogenous H_2_O_2_ is used to generate ROS, which reduces cell viability and increases intracellular oxidative stress levels [87], although ROS is mainly produced as byproducts of mitochondrial metabolism or is specifically generated by several oxidases, such as nicotinamide adenine dinucleotide phosphate oxidase (NOX) [88,89]. Oxidative stress increases the expression of ROCK2, which is involved in the main pathway through which TGF-β activates NF-κB [15]. p-Tyr42 RhoA, which is upstream of ROCK2 and the active form of RhoA, is translocated into the nucleus and elevates NOX expression, which results in an increase in ROS levels and senescence [90]. Exogenous H_2_O_2_ induces senescence and activates NF-κB translocation into the nucleus [91]. NF-κB is involved in senescence and induces the release of senescence-associated secretory phenotype (SASP) factors, including IL-1β, IL-6, IL-8, TNF-α, and MMPs [71], which contributes to the senescence of adjacent cells [92]. This study shows that MH4 ameliorates H_2_O_2_-induced mitochondrial depolarization and autophagy. ROS and oxidative stress are involved in the recruitment of mitophagy proteins [93]. Mitochondrial damage and loss of energy-generating capacity are characteristics of aged cells [79]. RhoA/ROCK2, which is activated by ROS, mediates mitochondrial functions, autophagy, and actin dynamics [26,94,95]. p-Tyr42 RhoA inhibition by MH4 attenuates this process.

In conclusion, MH4, a p-Tyr42 RhoA inhibitor, promotes the regeneration of hCECs and protects them against TGF-β- and H_2_O_2_-induced senescence via the ROS/NF-κB/mitochondrial pathway.

## Figures and Tables

**Table 1:** The differentially expressed genes of MH4-treated cells.

Gene_Symbol	Log_2_ (Fold Change)	*p*-Value
ZASP	−14.838634	0.01148286
GPAA1P2	−13.510711	0.00240992
DNAAF4	−13.311489	8.1673 × 10^−8^
SAXO1	−6.902963	0.01484039
MYOCD	−6.464285	0.0055486
PTGES3L-AARSD1	−6.338733	9.6461 × 10^−5^
ADORA2A	−5.373636	0.04736651
PATE2	−5.199964	0.02142765
MSH5	−4.751082	8.706 × 10^−6^
FAM24B-CUZD1	−4.147423	0.02095992
TIAF1	−3.838953	0.00654774
ARPC4-TTLL3	−3.791950	0.00029708
C8orf44-SGK3	−3.772787	9.2783 × 10^−5^
ISY1-RAB43	−3.712475	0.04409871
LOC105378797	−3.593747	0.045978
P2RX5-TAX1BP3	−3.218089	0.00027309
C5AR2	−2.878390	0.00391602
TIMM23B-AGAP6	−2.714220	2.1765 × 10^−7^
SLC25A21	−2.611541	0.02164787
NRK	−2.576926	0.0194514
DOK6	−2.542320	0.00346687
SLCO1C1	−2.479595	0.00214007
PCK1	−2.420558	0.01594034
TMEM63C	−2.297802	0.01672008
ETNPPL	−2.187381	0.03434799
HTD2	−2.133264	4.9329 × 10^−6^
SOX5	−2.123541	0.00893952
KIF15	−2.104287	0.04083181
SH3BGR	−2.064287	0.02573281
SLC25A18	−2.012335	0.02834259
HYI	2.044232	0.03988956
RN7SL1	2.079884	0.00071722
KLF2	2.194291	6.3451 × 10^−5^
ARHGAP5-AS1	2.329404	0.01048538
LRP2BP	2.349269	0.04300054
ZNF660-ZNF197	2.414622	0.01050856
SLC28A3	2.491094	0.02788002
VSTM5	2.679670	0.02360022
AMH	2.809669	0.03933912
ATRIP-TREX1	3.234381	9.2542 × 10^−5^
LRRC24	3.304932	0.00174921
MIR3648-2	3.799961	0.02673754
PPIP5K1P1	3.945428	0.00031678
GJA9-MYCBP	3.971132	0.01389741
DND1P1	3.995776	0.03677869
RPL12P16	4.074142	0.04330437
PLA2G4B	4.115890	0.04071125
NTAN1P2	4.394379	0.0286182
TMEM189-UBE2V1	5.555917	0.00099753
POC1B-GALNT4	5.621313	0.0364042
ZNF559-ZNF177	5.670612	0.00317011
FCF1P2	9.103486	0.03986722
STAG3L3	15.482174	0.04594955
RAD51L3-RFFL	16.348993	8.243 × 10^−8^
SETP17	17.898637	0.02859499
TVP23C-CDRT4	23.915473	0.01678133

**Table 2:** Molecular function of Gene Ontology by MH4.

	ID	Name	*p*-Value	FDR B and H	FDR B and Y	Bonferroni
1	GO:0008903	Hydroxypyruvate isomerase activity	6.030 × 10^−4^	2.291 × 10^−2^	1.126 × 10^−1^	4.583 × 10^−2^
2	GO:0015390	Purine-specific nucleoside:sodium symporter activity	6.030 × 10^−4^	2.291 × 10^−2^	1.126 × 10^−1^	4.583 × 10^−2^
3	GO:0015391	Nucleobase:monoatomic cation symporter activity	2.409 × 10^−3^	3.589 × 10^−2^	1.764 × 10^−1^	1.831 × 10^−1^
4	GO:0015389	Pyrimidine- and adenosine-specific:sodium symporter activity	2.409 × 10^−3^	3.589 × 10^−2^	1.764 × 10^−1^	1.831 × 10^−1^
5	GO:0005415	Nucleoside:sodium symporter activity	2.409 × 10^−3^	3.589 × 10^−2^	1.764 × 10^−1^	1.831 × 10^−1^
6	GO:0016861	Intramolecular oxidoreductase activity, interconverting aldoses and ketoses	3.611 × 10^−3^	3.589 × 10^−2^	1.764 × 10^−1^	2.744 × 10^−1^
7	GO:0015213	Uridine transmembrane transporter activity	3.611 × 10^−3^	3.589 × 10^−2^	1.764 × 10^−1^	2.744 × 10^−1^
8	GO:0005350	Pyrimidine nucleobase transmembrane transporter activity	4.212 × 10^−3^	3.589 × 10^−2^	1.764 × 10^−1^	3.201 × 10^−1^
9	GO:0015214	Pyrimidine nucleoside transmembrane transporter activity	4.812 × 10^−3^	3.589 × 10^−2^	1.764 × 10^−1^	3.657 × 10^−1^
10	GO:0005345	Purine nucleobase transmembrane transporter activity	6.012 × 10^−3^	3.589 × 10^−2^	1.764 × 10^−1^	4.569 × 10^−1^
11	GO:0005337	Nucleoside transmembrane transporter activity	6.012 × 10^−3^	3.589 × 10^−2^	1.764 × 10^−1^	4.569 × 10^−1^
12	GO:0005114	Type II transforming growth factor beta receptor binding	6.611 × 10^−3^	3.589 × 10^−2^	1.764 × 10^−1^	5.024 × 10^−1^
13	GO:0102545	Phosphatidyl phospholipase B activity	6.611 × 10^−3^	3.589 × 10^−2^	1.764 × 10^−1^	5.024 × 10^−1^
14	GO:0015205	Nucleobase transmembrane transporter activity	6.611 × 10^−3^	3.589 × 10^−2^	1.764 × 10^−1^	5.024 × 10^−1^

## Data Availability

The datasets are available from corresponding author on reasonable request.

## Supplementary Materials

The following supporting information can be downloaded at: https://www.mdpi.com/article/10.3390/antiox12061186/s1, Table S1: Primers for RT-PCR.
