# Supplementary material for: A p-Tyr42 RhoA Inhibitor Promotes the Regeneration of Human Corneal Endothelial Cells by Ameliorating Cellular Senescence
Source: Antioxidants (Basel). 2023 May 30;12(6):1186. doi: 10.3390/antiox12061186 (PMC10295357; doi:10.3390/antiox12061186)
Supplement: Supplementary file 1 [file antioxidants-12-01186-s001.zip › antioxidants-2373445-supplementary.pdf]

Table S1. Primers for RT-PCR

Gene	Primer	Primer sequence (5′ to 3′)
p21	Forward	CTGGGGATGTCCGTCAGAAC
	Reverse	CATTAGCGCATCACAGTCGC
H2Ax	Forward	CAACAAGAAGACGCGAATCA
	Reverse	CGGGCCCTCTTAGTACTCCT
CDKN2A	Forward	CATAGATGCCGCGGAAGGT
	Reverse	CTAAGTTTCCCGAGGTTTCTCAGA
BAX	Forward	CTGCAGAGGATGATTGCCG
	Reverse	TGCCACTCGGAAAAAGACCT
